# The impact of antidepressant treatment on population health: synthesis of data from two national data sources in Canada

**DOI:** 10.1186/1478-7954-2-9

**Published:** 2004-11-01

**Authors:** Scott B Patten

**Affiliations:** 1 Departments of Community Health Sciences and Epidemiology, University of Calgary, 3300 Hospital Drive NW, Calgary, Alberta, CANADA. T2N 4N1

## Abstract

**Background:**

In randomized, controlled trials, antidepressant medications have been shown to reduce the duration of major depressive episodes and to reduce the frequency of relapse during long-term treatment. The epidemiological impact of antidepressant use on episode duration and relapse frequency, however, has not been described.

**Methods:**

Data from two Canadian general health surveys were used in this analysis: the National Population Health Survey (NPHS) and the Canadian Community Health Survey (CCHS). The NPHS is a longitudinal study that collected data between 1994 and 2000. These longitudinal data allowed an approximation of episode incidence to be calculated. The cross-sectional CCHS allowed estimation of episode duration. The surveys used the same sampling frame and both incorporated a Short Form version of the Composite International Diagnostic Interview.

**Results:**

Episodes occurring in antidepressant users lasted longer than those in non-users. The apparent incidence of major depressive episodes among those taking antidepressants was higher than that among respondents not taking antidepressants. Changes in duration and incidence over the data collection interval were not observed.

**Conclusions:**

The most probable explanation for these results is confounding by indication and/or severity: members of the general population who are taking antidepressants probably have more highly recurrent and more severe mood disorders. In part, this may have been due to the use of a brief predictive diagnostic interview, which may be prone to detection of sub-clinical cases. Whereas antidepressant use increased considerably over the data-collection period, differences in episode incidence and duration over time were not observed. This suggests that the impact of antidepressant medications on population health may have been less than expected.

## Background

Depressive disorders are among the most important contributors to disease burden at the population level . While primary prevention for this condition has remained an elusive goal, provision of treatment has been viewed as having the capacity to reduce its impact on population health. Randomized, controlled clinical trials confirm that treatment with antidepressant medications can favorably impact the course of major depressive disorder. Clinical practice guidelines recommend acute treatment to reduce episode duration, continuation treatment to prevent relapse and maintenance treatment for those at high risk of recurrence [1].

Direct generalization of clinical trial data to the general population results in an expectation that depressive episode frequency and episode duration should be reduced in those receiving antidepressant treatment. However, outcomes reported in clinical trials are not necessarily reflected in "real world" outcome data. Since treatments are not assigned randomly in clinical settings, those treated are likely to have more severe illness (both in terms of episode duration and recurrence risk) than those who do not seek or receive treatment. In such circumstances, the effect of treatment may become confounded with the effect of the underlying disorder itself, and/or its severity [2].

Despite the plausible occurrence of confounding by indication and confounding by severity, population-based studies examining outcomes in relation to treatment status in the general population have not been reported. The objective of the current study was to estimate the incidence and duration of major depressive episodes in members of the general population who are receiving or not receiving antidepressant treatment.

## Methods

The National Population Health Survey (NPHS) is a longitudinal general health survey conducted by the Canadian government's statistical agency, Statistics Canada  The NPHS sample consists of 17,262 subjects selected in 1994 and 1995 (hereafter denoted 1994/95) who have been followed prospectively with interviews every two years since. Data has been released for the first four cycles (1994/95 to 2000/01). The Canadian Community Health Survey (CCHS) is another general health survey conducted by Statistics Canada. The CCHS has a very large sample size (n = 130,880) and employed a cross-sectional study design. Data collection for the CCHS occurred in 2000.

Both the NPHS and the CCHS utilized probability samples, based on the same sampling frame, from the Canadian general population. The sampling procedures incorporated both clustering and unequal selection probabilities. Valid inference therefore requires the use of sampling weights and statistical procedures accounting for non-independence within clusters. In order to deal with these methodological issues, a bootstrap procedure for variance estimation developed by Statistics Canada was employed in this analysis. This procedure accounts for the design effects.

Both the NPHS and the CCHS recorded medication use with a series of self-report items. The item relevant to this analysis asked whether the respondent had taken antidepressant medications during the month preceding the interview. Each interview also included the Composite International Diagnostic Interview Short Form for Major Depression (CIDI-SFMD), which was developed and validated by Kessler et al. [3]. This is a brief, fully structured instrument derived from a set of modified CIDI items. The CIDI-SFMD was designed to provide an operationalization of the DSM-IV [4] diagnostic criteria for major depression and is sufficiently brief that it can be included in general health surveys. The instrument detects symptoms indicative of major depression, and identification of five such symptoms (one of which must be depressed mood or loss of interest) indicates a high probability that the subject fulfilled DSM-IV criteria for major depression in the 12-months preceding the interview. It should be noted, however, that the Short Form does not contain all of the clinical significance probes and organic exclusion items that are included in the full CIDI, and may therefore detect some subclinical episodes [5]. A component of the CIDI-SFMD is an item that asks (of subjects reporting a probable episode of major depression) the number of weeks in the preceding year that were characterized by depressive symptoms.

In calculating incidence, pairs of observations across NPHS longitudinal data collection cycles were used. There were three suitable intervals covered by the four data collection rounds: 1994/95 to 1996/97, 1996/97 to 1998/99 and 1998/99 to 2000/01.

Attrition rates have generally been modest in the NPHS, with 76.6% of subjects having been successfully followed over the first four cycles [6]. In each instance, the subjects with major depression at the baseline interview were excluded and the remaining subjects were regarded as the population at risk of having a new or recurrent episode. It was not possible to differentiate between new and recurrent episodes, as lifetime history was not available. The proportion of subjects not reporting an episode in the 12-months preceding an interview who subsequently reported an episode in the 12-months preceding their interview 2 years later was used as an approximation of episode incidence. With the four available NPHS cycles, incidence could be estimated in this way across the three intervals.

## Results

**Figure 1:**
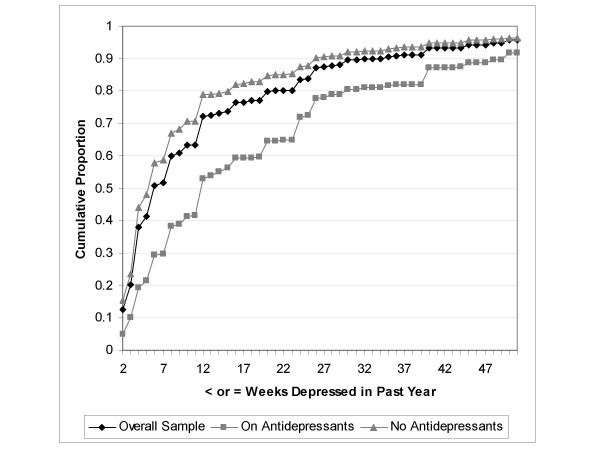
Cumulative proportion reporting ≤ specified number of weeks depressed in the past year for CIDI-SFMD positive subjects (n = 9508) in the Canadian Community Health Survey, by antidepressant use.

**Figure 2:**
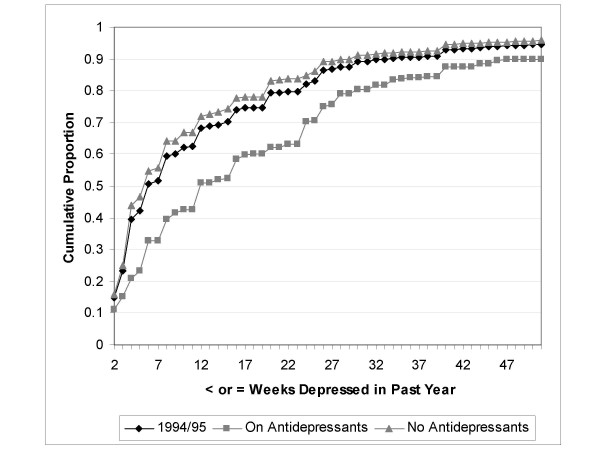
Cumulative proportion reporting ≤ specified number of weeks depressed in the past year for CIDI-SFMD positive subjects (n = 1030) in the 1994/95 National Population Health Survey, by antidepressant use.

Estimates of the number of weeks depressed in the past year derived from the CCHS were computed from 126,715 subjects who provided valid responses both to the CIDI-SFMD (including the duration item) and the antidepressant use survey item. There were 9729 subjects with an episode of major depression in the year preceding the interview and 9508 (97.7%) of these provided valid duration data. Figure 1 shows the number of weeks depressed in the past year as reported by subjects with major depression in the CCHS, depending on whether or not they were taking antidepressants. Weeks depressed in the past year is presented in Figure 1 as a cumulative frequency; the Figure depicts the proportion (on the 'y' axis) of subjects reporting a duration less than or equal to the number of weeks specified (on the 'x' axis). Figure 1 shows that subjects reporting antidepressant use had a greater number of weeks depressed in the year preceding the interview. Analogous plots were generated for the each of the NPHS cycles, and each presented a similar pattern. Figure 2 presents weeks depressed in the past year from the 1994/95 NPHS. In the CCHS, the mean reported number of weeks depressed in the past year was 10.8 weeks among those not taking antidepressants and 18.7 weeks among subjects who reported taking antidepressants.

**Figure 3:**
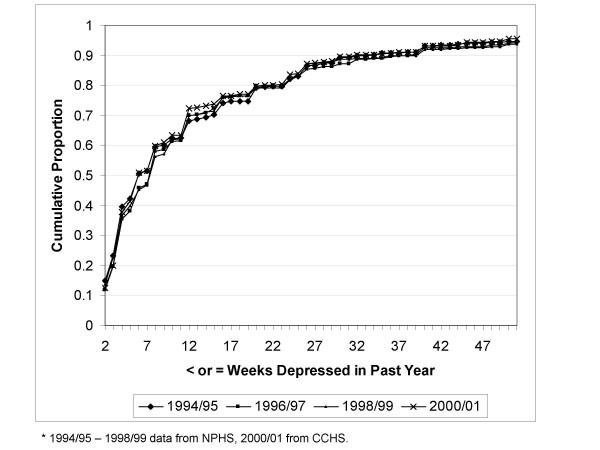
Proportion with ≤ specified weeks depressed in past year, by year of data collection.

Figure 3 presents the weeks depressed in the past year data from the NPHS and CCHS, without reference to antidepressant use. The reported weeks depressed in the past year is virtually identical in each of the four NPHS data collection cycles and the CCHS.

Table 1 presents approximate episode incidence, as calculated for the three intervals between the four NPHS data collection interviews. The point estimate for the 1996/97 to 1998/99 interval was slightly lower than the other two, but the confidence intervals associated with these estimates suggest that this difference could be due to chance. The incidence of new episodes in subjects reporting the use of antidepressant medication was approximately three times that of subjects not using antidepressants.

**Table 1:** Approximate incidence* in subjects taking or not taking antidepressants in the baseline year

Baseline year	Follow-up interview	Taking antidepressants		Not taking antidepressants	
		
		Approximate incidence* (unweighted proportion)	95% confidence interval	Approximate incidence* (unweighted proportion)	95% confidence interval
1994	1996	12.7% (20/172)	6.0 – 19.4	3.4% (277/9055)	2.9 – 4.0
1996	1998	7.8% (21/234)	3.5 – 12.0	3.5% (375/9127)	3.0 – 4.1
1998	2000	12.7% (47/352)	8.6 – 16.8	3.5% (317/8994)	2.9 – 4.0
Average		11.1%		3.5%	

*Proportion of subjects free from a major depressive episode in the year preceding the baseline interview who developed major depression in the year preceding the follow-up interview. These estimates are weighted to account for design effects. The unweighted raw proportions have been placed beside the weighted estimates.

## Discussion

Direct generalization of clinical trial data to the general population would lead to an expectation that subjects in the general population who report antidepressant treatment should have briefer episodes of major depression than those who do not receive such treatment. Similarly, one might hypothesize that subjects reporting antidepressant treatment should have a reduced incidence of episodes. In the current analysis of national survey data, neither expectation was found to hold true. Subjects reporting an episode of major depression and reporting receipt of antidepressant treatment reported more, rather than fewer, weeks depressed in the preceding year. Similarly, subjects who did not have an episode of major depression in the year preceding an interview but who nevertheless reported using antidepressant medications had a higher risk of having an incident episode than similar subjects who did not report taking antidepressants. The most obvious explanation for these findings is that of confounding by indication or severity.

Some of these results may have been due to inadequacies involving the data sources. One of these is the brief, and therefore somewhat crude [5] nature of the CIDI-SFMD as a measure of major depression. As this instrument does not contain detailed clinical significance probes, some sub-clinical episodes may have been detected. As the CIDI-SFMD does not contain organic exclusions, the instrument may have detected some episodes characterized by organic symptoms. The 12-month prevalence of major depression in the CCHS was 7.4% which is higher than most recently published estimates of major depression prevalence, consistent with the possibility of non-specificity. This draws into question whether these results would be replicated with the use of a more detailed diagnostic instrument. However, this concern should not be overstated. Some studies using the full CIDI have reported higher estimated 12-month prevalence [7,8]. Severe depressive disorders that are treated with antidepressants may have longer episode durations and higher relapse rates than less severe and untreated disorders.

Items evaluating antidepressant use in each survey referred to use of the medications during the past month, whereas probable major depressive episodes occurring during the past year are detected by the CIDI-SFMD. Therefore, even though subjects who reported taking antidepressants were found to be more likely to have a subsequent episode of major depression, the data did not allow a determination of whether some of these subjects may have stopped or started antidepressants at some time during the follow-up interval.

Another limitation of the data sources used in this project was the lack of comorbidity data. Many of the subjects taking antidepressants may have been doing so for indications such as the prophylaxis of migraine headaches or for treatment of anxiety disorders. Both migraine headaches [9,10] and anxiety disorders [11] are frequently associated with major depression. To the extent that these disorders impact upon the risk and prognosis of major depressive episodes, they could also confound associations between antidepressant use, major depression episode incidence and episode duration.

While confounding by indication and severity offer, perhaps, the most appealing explanation for these results it is important to emphasize that the attractiveness of these explanations is based on a set of assumptions: that antidepressants are efficacious in reducing episode duration and the risk of relapse and recurrence. An altered set of initial assumptions leads to other possible interpretations. For example, some authors have hypothesized that antidepressant treatment may lead to a deterioration in the long-term course of mood disorders [12]. This hypothesis, although not widely accepted, predicts that episode duration and incidence would be higher in those reporting antidepressant use than in those not using these medications. A finding consistent with this idea was a meta-analysis by Baldessarini et al., which found that subjects taking antidepressant medications for longer periods were more likely to relapse upon discontinuation [13].

Generally, the public health challenges associated with major depression have been conceptualized in "common sense" terms, and in a way that does not depend on underlying etiological features of the disorder. For example, the idea that preventing relapse (episode incidence) should translate into reduced prevalence and reduced disease burden seems on the surface to be a common-sense belief. However, it has been hypothesized that depression can assume an adaptive role [14]. If an adaptive role for depression involves, for example, limiting an individual's interaction with a stressful environment (e.g. if depressive symptoms serve the adaptive purpose of discouraging interaction with elements of the environment that trigger them), then treatment of depression may indirectly lead to increased stress exposure. If this is true, then antidepressant treatment may increase people's comfort and capacity to function in a stressful environment, but may also increase the intensity of stress in the environment that surrounds them. Such complex dynamics could obscure an expected decline in prevalence due to increased treatment.

## Conclusions

Most of the literature concerned with major depression and public health has emphasized that the disorder is under-treated, although more recent studies have begun to move beyond the frequency of treatment to address the issue of treatment adequacy [15]. There is an emerging need to address the lack of epidemiological evidence confirming the population-health benefits of increased antidepressant treatment. Specifically, it will be necessary to determine the extent to which negative outcomes in association with antidepressant treatment in observational data sources represent a methodological artifact.

## List of Abbreviations

CIDI-SFMD Composite International Diagnostic Interview Short Form for Major Depression

DSM-IV Diagnostic and Statistical Manual of Mental Disorders, Fourth Edition.

MDE Major Depressive Episode

NPHS National Population Health Survey

CCHS Canadian Community Health Survey (iteration 1.1)

## Competing Interests

The author(s) declare that they have no competing interests.
